# Ethnomedicinal herbs in African traditional medicine with potential activity for the prevention, treatment, and management of coronavirus disease 2019

**DOI:** 10.1186/s43094-021-00223-5

**Published:** 2021-03-20

**Authors:** Olutayo Ademola Adeleye, Mbang Nyong Femi-Oyewo, Oluyemisi Adebowale Bamiro, Lateef Gbenga Bakre, Akinyinka Alabi, Joseph Senu Ashidi, Olalekan Adeyinka Balogun-Agbaje, Oluwakemi Mary Hassan, Gbemisola Fakoya

**Affiliations:** 1 Department of Pharmaceutics and Pharmaceutical Technology, Federal University Oye Ekiti, Oye-Ekiti, Ekiti State Nigeria; 2 Department of Pharmaceutics and Pharmaceutical Technology, Olabisi Onabanjo University, Ago-Iwoye, Ogun State Nigeria; 3 Department of Pharmacology, Olabisi Onabanjo University, Ago-Iwoye, Ogun State Nigeria; 4 Department of Plant Science, Olabisi Onabanjo University, Ago-Iwoye, Ogun State Nigeria; 5 Department of Pharmaceutical Microbiology, Olabisi Onabanjo University, Ago-Iwoye, Ogun State Nigeria; 6 Department of Pharmacology, University of Lagos, Lagos, Lagos State Nigeria

**Keywords:** Coronavirus, SARS-CoV-2, COVID-19, Traditional medicine, Ethnomedicinal herbs

## Abstract

**Background:**

Ethnomedicine, a study of traditional medicine, is significant in drug discovery and development. African traditional medicine has been in existence for several thousands of years, and several drugs have been discovered and developed from it.

**Main text:**

The deadly coronavirus disease 2019 (COVID-19) caused by a novel coronavirus known as SARS-CoV-2 has widely spread globally with high mortality and morbidity. Its prevention, treatment and management still pose a serious challenge. A drug for the cure of this disease is yet to be developed. The clinical management at present is based on symptomatic treatment as presented by individuals infected and this is by combination of more than two drugs such as antioxidants, anti-inflammatory, anti-pyretic, and anti-microbials. Literature search was performed through electronic searches of PubMed, Google Scholar, and several research reports including WHO technical documents and monographs.

**Conclusion:**

Drug discovery from herbs is essential and should be exploited for the discovery of drugs for the management of COVID-19. This review is aimed at identifying ethnomedicinal herbs available in Africa that could be used for the discovery and development of a drug for the prevention, treatment, and management of the novel coronavirus disease 2019.

## Background

Ethnomedicine is a study of traditional medicine involving bioactive compounds of plants and animals origin from diverse cultural groups. It comprises ethnobiology, ethnobotany, and ethnopharmacology. The significance of ethnomedicinal study is for drug discovery. Scientific ethnomedicinal studies have been used and adopted as a source of lead compound identification in drug discovery and development processes [1, 2].

Traditional medicine is the “sum total of the knowledge, skills, and practices based on the theories, beliefs, and experiences indigenous to different cultures, whether explicable or not, used in the maintenance of health as well as in the prevention, diagnosis, improvement, or treatment of physical and mental illness” [3]. African traditional medicine is highly dependent on cultural, religious, and spiritual belief. It is as old as the world and still in existence even after the introduction of science-based medicine by the Europeans [4].

The causative agent of the deadly coronavirus disease 2019 (COVID-19) is a novel coronavirus known as SARS-CoV-2. COVID-19 outbreak was declared as a pandemic by the World Health Organization on 11 March 2020 due to the fast, wide spread and severity of the disease in 114 countries globally. The treatment and management of the disease has posed a serious challenge. Various studies on how to mitigate the scourge of this pandemic is in progress. Studies on the use of ethnomedicinal herbs in the prevention, treatment, and management of COVID-19 are obtainable in literatures [5–7].

The clinical management of COVID-19 generally involves treatment of the symptoms associated with the disease usually involving the combination of more than two drugs such as antioxidants for the reduction of oxidative stress which may cause injury to the lung cells [8], anti-inflammatory agents for the reduction of inflammation due to rapid viral replication and cell infiltration [9], anti-pyretic agent for reduction of fever [10], anti-microbials for the reduction and treatment of opportunistic infections due to reduced immunity [11], anti-viral for inhibition of viral entry and reduction in viral replication [12], nutritional vitamins, and minerals like ascorbic acid and zinc to reinforce the immune system [13, 14].

Literature search was performed to identify and extract relevant information through electronic searches of PubMed, Google Scholar, and several research reports including WHO technical documents and monographs.

This review attempts to identify ethnomedicinal herbs (with their vernacular names for ease of identification) available in Africa that could be used to develop drugs for the prevention, treatment and management of the novel coronavirus disease 2019. Some of these plants are not indigenous to Africa but have been cultivated for their beneficial values. It is necessary to source for these herbs locally to reduce cost of research and development and for reproducibility and sustainability.

## Main text

*Abrus precatorius* Linn. Gaertn

Family: Fabaceae

Common names: Rosary pea, Jequirity, Crab’s eyes

Local names: Mongaluchi (Swahili, Kenya); Mutiti (Lozi, Zambia) Ndela (Chagga, Tanzania); Mantumbi (Badyara, Senegal); Amabope (Ndebele, South Africa); nsimani (Tsonga, South Africa); Iwere-jeje, Ojuologbo (Yoruba, Nigeria); Anya nnunu (Igbo, Nigeria), Da marzaya (Hausa, Nigeria) [15–17].

Bioactive compounds: Abrine, Abrectorin, Abrusoside A, Abricin, Abraline, Choline, Glycyrrhizin, Luteolin, Trigonelline [18, 19].

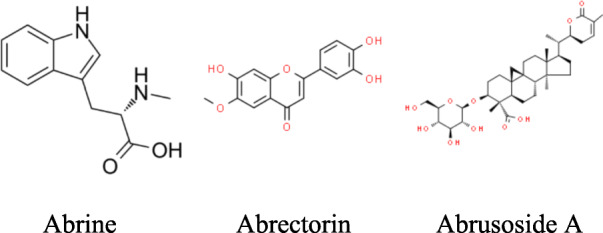



*Abrus precatorius* is an herbaceous, perennial, flowering climbing plant. It is indigenous to Africa, Asia, and Australia but now naturalized in many countries. The plant is widely distributed throughout Africa. The leaves are used in traditional medicine for the treatment of cough, cold, fever, conjunctivitis, pains, constipation, yellow fever, tuberculosis, and other pulmonary problems; the seeds are used as contraceptives and for the treatment of eye inflammation and joint pains; the stem bark and roots are used for the treatment of diabetes, venereal diseases, bacterial, and fungi diseases and as sedatives [15, 20].

Some of the pharmacological activities of *Abrus precatorius* that have been reported are anti-convulsant, anti-asthmatic, anti-inflammatory, analgesic, anti-arthritic, anti-rheumatic, anti-diabetic, anti-oxidant, anti-depressant, anti-microbial, and anti-viral activity [19, 21, 22].

The leaf and root of *Abrus precatorius* would be a potential source of anti-coronavirus drug since it contains glycyrrhizin as documented by [19, 23]. Several studies reported the activity of glycyrrhizin against coronaviruses [24–26].

*Achyranthes aspera* Linn.

Family: Amaranthaceae

Common names: Devil’s horsewhip, Prickly chaff flower

Local names: Isinama (Zulu South Africa); Moxato (Botswana); Bhomane (Lesotho); Udombo (Zimbabwe); Turura (Swahili, Kenya); Vatofosy (Madagascar); Epa aboro (Yoruba, Nigeria); Odụdu ngwele (Igbo, Nigeria) [27, 28].

Bioactive compounds: Ecdysterone, Eugenol, Betaine, Triacontanol [29].

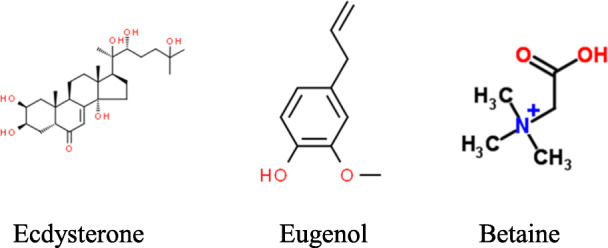



*Achyranthes aspera* is an erect perennial herbal weed. The origin of the plant is not known but it is believed to be indigenous to either South Asia or/and Africa. It is widely naturalized in the tropics and sub-tropics. All the plant parts (leaves, seeds, roots, and shoots) are used in African traditional medicines for the treatment of malaria, ulcer, fever, arthritis, diarrhea, dysentery, hemorrhoids, itching, and headache [28]. It is also used as an antioxidant, anti-inflammatory agent, expectorant, diuretic, and inhalation for respiratory problems such as pneumonia and asthma [30, 31].

*Achyranthes aspera* possesses weak anti-viral activity [32]; however; it has good antioxidant and anti-inflammatory properties. It is rich in ascorbic acid which confers the antioxidant property that boosts immunity against infections like SARS-coronavirus [33, 34]. The anti-inflammatory properties could be beneficial in alleviating inflammation due to SARS-coronavirus [28, 35]. *Achyranthes aspera* will be effective in the management of COVID-19.

*Allium sativum* L.

Family: Liliaceae

Common names: Garlic

Local names: Thuum (Arabic, Egypt); Ivimbampunzi (Xhosa, South Africa); Kitunguu-saumu (Swahili, Tanzania); Tafarnuwa Hausa, Nigeria); Ayo-ishi (Igbo, Nigeria); Ayuu (Yoruba, Nigeria) [36–38].

Bioactive constituents: Ajoene, Allicin, Diallyl disulfide, Vinyldithiins [39, 40].

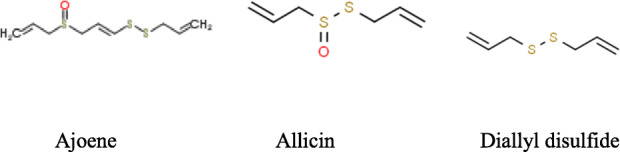



*Allium sativum* is a monocotyledonous erect flowering plant native to central Asia. The plant is now widely cultivated and distributed all over the world. The part of the plant mainly utilized in traditional medicine in Africa and in other countries is the bulb. It has been used as a remedy in the past during epidemics such as amoebic dysentery, cholera, diphtheria, tuberculosis, and influenza in Egypt [41]. It is used in African traditional medicine in the treatment of skin diseases, intestinal disorders, respiratory diseases, bacterial infections, worm infestation, and tumors [42].

Pharmacological activities of garlic as highlighted in scientific literatures include anti-diabetic, anti-inflammatory, antioxidant, hepatoprotective, cardiovascular, anti-bacterial, anti-fungal, anti-viral, and anti-cancer activity [40, 42, 43].

Keyaerts et al. reported *Allium sativum* of possessing marked antiviral activity against coronaviruses [44]. Thuy et al. suggested *Allium sativum* to be a valuable source of anti-SARS-CoV-2 [45].

*Annona muricata* Linn.

Family: Annonaceae

Common names: Soursop, Graviola, Prickly custard apple

Local names: Soursap (Krio, Sierra Leone); Soursapi (Mende, Sierra Leone); Omusitafeli (Basoga Uganda); Ekitafeli (Baganda Uganda); Araticum (Benin); Sabasaba, Ebom beti (Cameroon); Apre (Ghana); Corossol (Madagascar); Ebo, Apekan (Yoruba, Nigeria); Fasadarur or Tuwon biri (Hausa, Nigeria); Sawansop (Ibo, Nigeria) [46–50].

Bioactive compounds: Annonaceous acetogenins-Annocatalin, Annomuricin A, Annocatacin, Muricatocin [51].

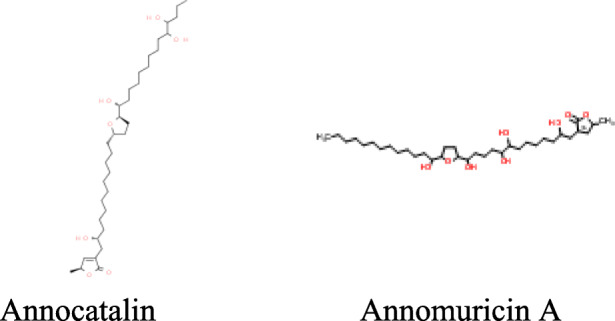



*Annona muricata* is a perennial small woody evergreen tree considered to be native to North and South America. The plant is widely found in East and West Africa as cultivar. The leaf, back, root, and fruit have been in use for decades in African traditional medicine. The leaves are used as anti-inflammatory agent [52], and also used to treat diabetes, headaches, rheumatism, and insomnia [53]. The ground seeds are used to treat coughs, pain, and skin diseases [51].

*Annona muricata* has been reported to possess anti-microbial and anti-viral activity [54]. The seed is reported to possess anti-SARS coronavirus activity. Oyebamiji et al. discovered that *Annona muricata* seed has a promising ability to inhibit SARS coronavirus [55]. Trivedi et al. also highlighted that the seeds of *Annona muricara* can be used to inhibit COVID-19 pathway [56].

*Artemisia afra* Jacq. ex Willd.

Family: Asteraceae

Common names: Wild wormwood, African wormwood

Local names; Wilde-als (Afrikaans, South Africa); Umhlonyane (Xhosa, South Africa and Zimbabwe); Mhlonyane (Zulu, South Africa); Lengana (Zimbabwe); Nyumba (Luo, Kenya); Ariti (Amharic, Ethiopia) [57].

Bioactive compounds: Acacetin, Scopoletin, Yomogiartemin, Dihydroxybishopsolicepolide [58, 59].

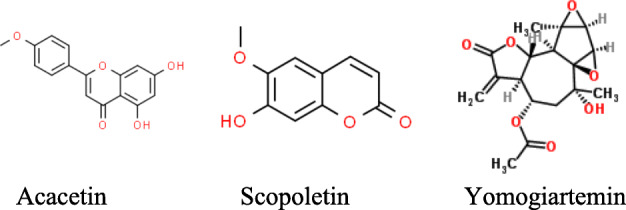



*Artemisia afra* is a perennial woody shrub, a specie of *Artemisia* which is indigenous to Africa. It originates and widely distributed in South Africa, spreading to some countries in North and East Africa. The roots, stems, and leaves are used in African traditional medicine to treat coughs, respiratory diseases, fevers, asthma, malaria, colds, chills, gout, diabetes, influenza, and convulsions [59, 60]. Studies on the activity of the plant on viruses such as HIV and influenza have been reported in scientific publications [61, 62].

The traditional use of *Artemisia afra* to treat coughs, respiratory diseases like asthma, whooping cough, and bronchitis prompted researchers to carry out scientific studies on the plant to ascertain the claim of traditional practitioners. Some studies highlighted and established the activity of the plant as claimed by traditional practitioners [63, 64]. Since *Artemisia afra* has been established to possess activity for the treatment of respiratory diseases, it could be beneficial as a supplement in the management of SARS-coronavirus. Although there is no record in the literature which indicates that *A. afra* possesses activity against coronaviruses but it could be used to support the management of COVID 19.

A specie of Artemisia known *Artemisia annua* found in Asia has some documented scientific evidences of activity against coronaviruses [65, 66]. This plant is now widely cultivated is some countries in Africa especially East Africa (Kenya, Uganda, Tanzania, Ethiopia, Mozambique, Madagascar) [67].

*Azadirachta indica* A. Juss.

Family: Meliaceae

Common name: Neem

Local names: Aforo-oyinbo (Yoruba, Nigeria); Aku shorop, Ogwu iba, Ogwu akom (Igbo, Nigeria); Dogonyaro (Hausa, Nigeria); Nimtso (Krobo, Ghana); Kingtsho (Ga, Ghana); Dua gyare (Ashanti, Ghana); Liliti (Ewe, Ghana); Kinitsi (Togo); Mwarubaini, Mkilifi (Swahili, Kenya and Tanzania) [17, 68–70].

Bioactive compounds: Azadirachtin, Nimbin, Nimbolide, Nimbidin, Nimbidol [71, 72].

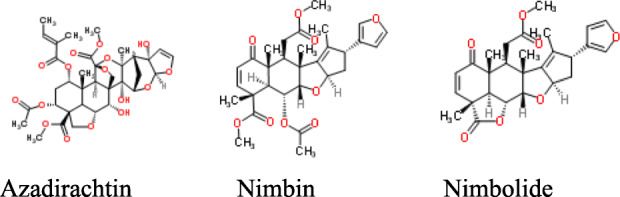



*Azadirachta indica* is an evergreen drought-resistant woody plant native to India and now widely seen growing in West and East Africa. Every part of the plant (stem, leaves, bark, roots, seeds, and flowers) has been used in traditional medicine in Nigeria and other countries like India to cure different conditions such as malaria, headache, stomach ulcers, jaundice, anemia, dental problems, bacterial, fungi, and viral infections [68, 73].

Anti-viral activity of *Azadirachta indica* has widely been reported on some viruses (apart from coronaviruses) such as coxsackieviruses, dengue virus, and hepatitis C virus [72, 74]. Recently, scientists have reported anti-viral activity of *Azadirachta indica* against coronaviruses as a result of the urgency required for the development of a specific anti-viral drug for the prevention, treatment, and management of COVID-19. Pooladanda et al. [75]; Borkotoky and Banerjee [76]; Ather and Costigliola [77] reported possible beneficial effect of *Azadirachta indica* in the treatment of SARS-CoV-2 infections.

*Cryptolepis sanguinolenta* (Lindl.) Schltr

Family: Asclepiadaceae

Common names: Ghana quinine, Yellow dye root

Local names: Nibima (Twi, Ghana); Kadze (Ewe, Ghana); Kɔli mekari (Bantu, Kenya); Paran pupa (Yoruba, Nigeria); Akpaoku (Igbo, Nigeria); Gangamau (Hausa, Nigeria); Ouidoukoi (Bambara, Mali) [78–80]..

Bioactive compounds: Cryptolepicarboline, Cryptolepine, Cryptospirolepine, Cyptomisrine, Cryptoquindoline [81].

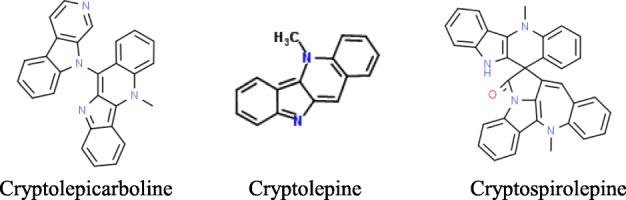



*Cryptolepis sanguinolenta* is a thin-stemmed, climbing, twining perennial flowering shrub. It is indigenous to Central, Eastern, and Western African regions. The root is used in African traditional medicine for the treatment of malaria, jaundice, hepatitis, hypertension, stomach and intestinal disorders, urinary and upper respiratory tract infections, rheumatism, amoebic dysentery, diarrhea, and venereal diseases [78, 82, 83]. The pharmacological activities of the plants are anti-plasmodial, anti-cancer, anti-fungal, anti-bacterial, anti-viral, hypotensive, anti-pyretic, anti-inflammatory, and anti-hyperglycemic activities [81, 84].

*Cryptolepis sanguinolenta* anti-viral activity on herpes implex virus 1 and 2 was reported by Buhner 2013 [85]. Gyebi et al. highlighted the plant as a potential inhibitor of coronavirus [86].

*Curcuma longa* Linn.

Family: Zingiberaceae

Common names: Turmeric

Local names: Mandano or Manjano (Swahili, Kenya); kurkum (Arabic, Somalia, Egypt); Ata ile pupa (Yoruba, Nigeria); Gangamau (Hausa, Nigeria); Nwandumo, Ohu boboch (Igbo, Nigeria) [87, 88].

Bioactive constituents: Curcumin, Quercetin, Curcuminoids [89, 90].

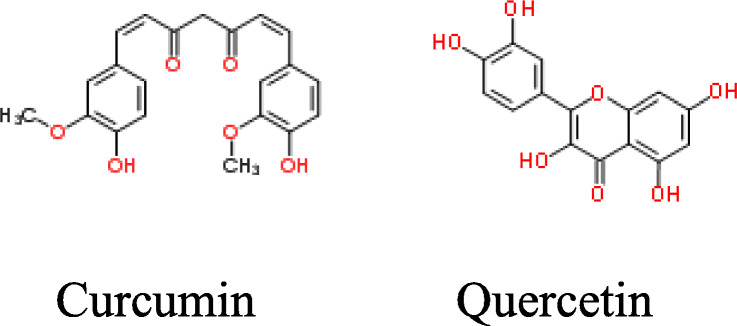



*Curcuma longa* is a rhizomatous, perennial, small flowering herbaceous plant indigenous to South Asia. The plant is widely cultivated in many parts of East and West Africa and other continents for its culinary spicy and medicinal value. The rhizome is the most commonly used part in Africa for the traditional treatment of some ailments such as headache, skin diseases, jaundice, smallpox, microbial infections, diarrhea, diabetes, arthritis, anorexia, cough, sinusitis, conjunctivitis, and diabetic wounds [17, 91].

The pharmacological activities of *Curcuma longa* has been extensively studied and found to possess anti-inflammatory, anti-ulcer, antioxidant, anti-diabetic, anti-coagulant, anti-fertility, anti-neoplastic, anti-microbial, anti-viral, wound healing, cardiovascular protective, hepatoprotective, and immunostimulant activity [92, 93].

The plant has also been studied as a potential source of SARS coronavirus treatment, prevention, and management. Wen et al. [94] and Zahedipour et al. [95] reported *Curcuma longa* as a plant with potent anti-viral activity against SARS coronaviruses, so it could be effective in the treatment of SARS-CoV-2. Lin and Ying [96] exploited the antioxidant and anti-inflammatory activity of curcumin for the treatment of pneumonia in patients as a result of COVID-19 infection.

*Euphorbia hirta* Linn.

Family: Euphorbiaceae

Common names: Hairy spurge, Garden spurge, Milkweed, Asthma-plant.

Local names: Rooi euphorbia (South Africa); Makore selu (*Badyara, S*enegal); ku tim (Diola-flup, Senegal); Fuŋkele (Limba, Sierra Leone); kakaweadwe (Akan-Asante, Ghana); Ahinkogye (Twi, Ghana); Akubaa (Nzema, Ghana); Noonon Kurciyaa (Hausa, Nigeria); Obụ Anị, Oba Ala, Udani, ogwu ngwo (Igbo, Nigeria); Akun Esan, Buje, Ege-Ile, Emi-Ile (Yoruba, Nigeria) [97, 98].

Bioactive compounds: Rutin, Euphorbin E, Kaempferol, Afzelin, Quercitrin, Myricitrin, Choline, Camphol [99].

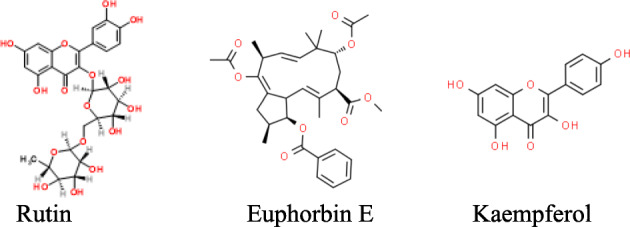



*Euphorbia hirta* is a small annual hairy weed that is native to tropical America, and now widely spread to the tropics and subtropics. All the plant parts are widely used in African traditional herbal medicine for the treatment of wounds, boil, diarrhea, dysentery, respiratory and bronchial disorders, and malaria [17, 98].

*Euphorbia hirta* has been reported to possess anti-malarial, anti-helmintic, anti-asthmatic, anti-spasmodic, anti-fertility, sedative, wound healing, anti-bacterial, and anti-fungal properties [99, 100]. The plant was reported to possess anti-viral activity against HIV-1 and HIV-2 [101].

*Euphorbia hirta* has no anti-SARS-coronavirus activity but has good activity on respiratory problems which is a major symptom of coronavirus. Shahrajabian et al. listed it as one of the most important herb used for the treatment of respiratory diseases [102]. So, it can be used to support the treatment and management of COVID-19 patients. Onyeji highlighted in his study that *Euphorbia Hirta* can alleviate some of the respiratory symptoms associated with COVID-19 [103].

*Garcinia kola* Heckel

Family: Clusiaceae

Common names: Bitter kola

Local names: Tweapia (*Anyi*, *Ghana);* Akuilu (Igbo, Nigeria); Orogbo (Yoruba, Nigeria); Namijin goro (Hausa, Nigeria) [104, 105].

Bioactive constituents: Kolanone, Kolaflavanone, Garcinoic acid, Kolaviron, and Garciniflavanone [105, 106].

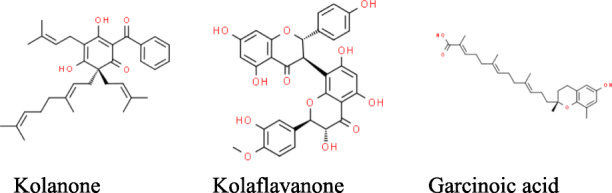



*Garcinia kola* is an evergreen, perennial, medium-sized flowering tree that is indigenous to Central and Western Africa especially Benin, Cameroon, Congo, Ivory Coast, Ghana, Liberia, Nigeria, and Senegal where they are mostly found and used. Every part of the plant (leaves, fruits, seeds, stems, barks, twigs, and roots) is used by African traditional medical practitioner in the treatment of various diseases such as bronchitis, throat infection, skin infection, headache, stomach ache, gastritis, cold, cough, malaria, tuberculosis, typhoid fever, malignant tumors, gonorrhea, fresh wounds, liver disorders, and jaundice [107, 108].

The pharmacological activities of the plant include anti-inflammatory, anti-oxidant, anti-asthma, anti-arthritis, anti-ulcer, anti-hypertensive, anti-microbial, anti-viral, anti-diabetic, and anti-hepatotoxic activities [106, 109].

Oladele et al. [110] observed that *Garcinia kola* has anti-SARS-CoV-2 inhibitory potential while Ikpa et al. [111] listed it as a plant with promising result against coronaviruses.

G*lycyrrhiza glabra* L.

Family: Fabaceae

Common names: Licorice

Local names: Susu (*Swahili*, Kenya); Dhalashada (Somali, Somalia); Irkessus (Arabic, Egypt); Ewe omisinmisin (Yoruba, Nigeria) [112, 113].

Bioactive constituents: Glycyrrhizin, Licoriphenone, Glycyrrhizic acid, Prenyllicoflavone A, etc. [114].

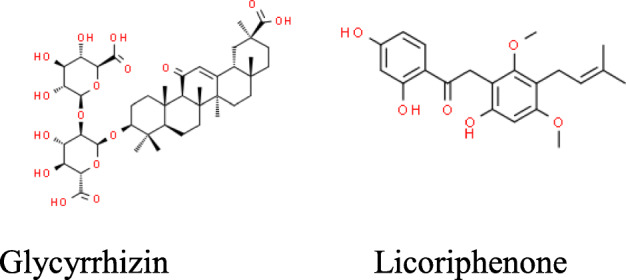



*Glycyrrhiza glabra* is an herbaceous perennial herb native to south-western Asia and southern Europe but widely cultivated for commercial purposes in North Africa (Egypt) and South Africa. The roots and rhizomes are used in ancient Egypt to treat upper respiratory disease like common cold, cough, bronchitis, and sore throats. It is used to treat heartburns and skin diseases [115].

G*lycyrrhiza glabra* possesses some pharmacological activities which includes anti-ulcer, anti-inflammatory, antioxidant, anti-hyperglycemic, anti-allergic, anti-cancer, anti-malarial, memory-enhancing, anti-microbial, and anti-viral activity [116, 117].

The anti-coronavirus activity of G*lycyrrhiza glabra* has been extensively studied and reported to be as a result of the presence of glycyrrhizic acid and glycyrrhizin [114, 118]. Several reports displaying anti-SARS-CoV activity of G*lycyrrhiza glabra* are highlighted in literatures. Hoever et al. confirmed in their research that licorice possesses anti-SARS-CoV [25].

In a recent study on COVID-19, G*lycyrrhiza glabra* was reported to successful inhibit SARS-CoV replication and was recommended for the management COVID-19 [25, 66, 119, 120].

*Moringa oleifera* Lam.

Family: Moringaceae

Common names: Horseradish tree, Drumstick

Local names: La-Banyu (Bwaba, Burkina Faso); Atiuwuse (Ewe, Ghana); Anamambo (Malagasy, Madagascar); Neverday (Wolof, Senega); Zagalanda (Tonga, Zambia); Mlonge (Swahili, Kenya, and Tanzania); Al-ruwag (Arabic, Sudan); Ewe ile, Ewe igbale (Yoruba , Nigeria); Odudu oyibo, Okwe oyibo (Igbo, Nigeria); Zongallagandi, Bagaruwar masar (Hausa , Nigeria) [121–123].

Bioactive constituents: Apigenin, Luteolin, Phytosterols, Quercetin, Terpenoids, Caffeic acid [124–126].

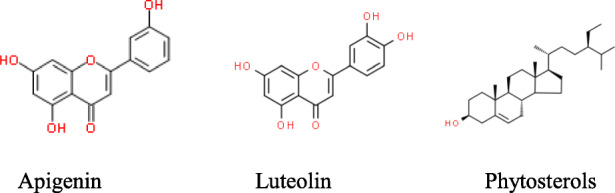



*Moringa oleifera* is a fast-growing drought-resistant, deciduous, perennial softwood tree which is native to India but now widely cultivated and naturalized in Africa and many other tropical and subtropical countries for variety of uses such as food and traditional herbal medicine. All the parts of the plant are used traditionally in many African countries for bone setting and enhancement of lactation. They are also used in the treatment of impotence, heartburn, asthma, flu, cough, pneumonia, common cold, bronchitis, syphilis, malnutrition, diabetes, hypertension, gastric ulcers, malaria, and fever [127, 128].

The pharmacological activities of *Moringa oleifera* are numerous and includes, analgesic, anti-inflammatory, local anesthetic, anti-allergic, anti-microbial, antioxidant, anti-cancer, cardiovascular, gastroprotective, hepatoprotective, neuroprotective, anti-ulcer, diuretic, anti-helmintic, hypoglycemic, blood lipid-reducing, immunomodulatory, and anti-diarrheal activity [124, 125, 129].

*Moringa oleifera* possesses some anti-viral activities but not on coronaviruses [130, 131]. However, they could be used to complement and supplement the management of SARS-CoV diseases because of its richness in minerals (zinc, potassium, calcium, magnesium), and vitamins (vitamin C) [132, 133]. Adejuwon et al. reported an herbal mixture formulation containing *Moringa oleifera* to possess SARS-CoV-2 inhibitory activity [134].

*Nigella sativa* L.

Family: Ranunculaceae

Common names: black seed, black cumin, fennel flower

Local names: Habbah Sawda’ or ‘Habbatul Barakah’ (Arabic, Egypt); Tikur azmud (Amharic, Ethopia); Habatu Sauda (Hausa, Nigeria); Asofeyeje (Yoruba, Nigeria ) [135, 136].

Bioactive compounds: Thymoquinone, Cymene, Carvacrol, Thymohydroquinone, Dihydrothymoquinone, thymol [137].

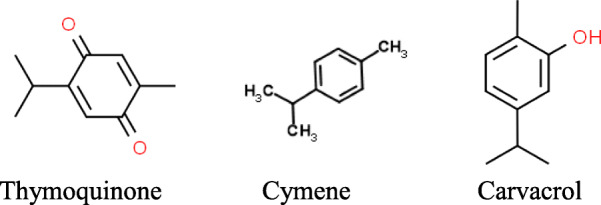



*Nigella sativa* is an annual flowering plant native to North Africa and some other regions like Eastern Mediterranean, the Indian subcontinent, and Southwest Asia. The seed of the plant has been in use for centuries in Africa and across many continents. It is widely used traditionally to treat asthma, cough, bronchitis, rheumatoid arthritis, diabetes, and hypertension, and to boost the body’s immune system to fight illness [138]. It possesses pharmacological properties such as anti-inflammatory, anti-cancer, analgesic, antioxidant, anti-microbial, anti-parasitic, and anti-viral properties [139, 140].

Ulasli et al. reported a decrease in the replication of Coronavirus with ethanol extract of *Nigella sativa* seed [141]. There are more recent studies which showed that *Nigella sativa* possesses potential anti-coronavirus activity [142–144].

*Psidium guajava* Linn.

Family: Myrtaceae

Common names: Guava

Local names: Koejawal (Afrikaans, South Africa), gouyav (Seychelles Creole, Seychelles),

Guava (Hausa, Nigeria); Gurfa (Yoruba, Nigeria); Gwaibwa (Igbo, Nigeria); Mupeera (Luganda, Uganda); Biabo (Mandinka, Mali); Mpera (Swahili, Tanzania, and Kenya); Zeitun, (Tigrigna, Eritrea) [145, 146].

Bioactive compounds: Ascorbic acid, Caryophyllene, Gallic acid [147].

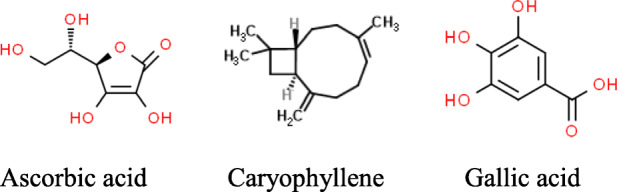



*Psidium guajava* is a perennial, shallow-rooted evergreen small tree; it is believed to be native to tropical America but now naturalized and widely cultivated and distributed in almost all African countries and other tropical and subtropical countries. The leaf and bark of the plant have been used for several decades in African traditional medicine. It is used for the treatment of malaria in South Africa, Nigeria, and Tanzania; treatment of hypertension and diabetes in Togo and Nigeria; tuberculosis in Nigeria; HIV in Tanzania; and bacterial infection in South Africa and Guinea [148–150].

Some of the pharmacological activities of *Psidium guajava* that are reported include anti-bacterial, anti-fungal, anti-hypertensive, anti-cancer, anti-inflammatory, antioxidant, immune-system stimulatory, anti-diabetic, and anti-plasmodial activities [151].

*Psidium guajava* was reported by Fukumoto et al. in a Taiwanese patent in 2010 to possess Anti-SARS coronavirus activity [152]. Some studies have been carried out showing that *Psidium guajava* has some potential bioactive compounds that can breakdown coronavirus proteins [126, 153, 154].

*Zingiber officinale* Roscoe

Family: Zingiberaceae

Common names: Ginger

Local names: Gnamakou (Dioula, Burkina Faso); Akakaduro (Akan, Ghana); Didière (Wolof, Senegal); Tangawizi (Swahili, Tanzania); Citta (Fulfulde, Nigeria); Citaraho (Hausa, Nigeria); Jinja (Igbo, Nigeria); Atale (Yoruba, Nigeria) [155–157].

Bioactive constituents: Zingiberene, Zingerone, Gingerol, Gingerdiol, Shogaol, Paradols, Curcumene, etc. [158, 159].

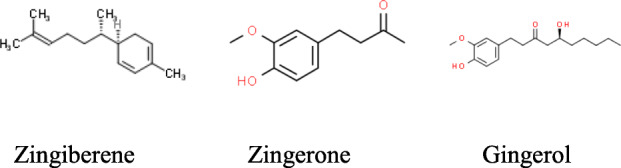



*Zingiber officinale* is a rhizomatous, perennial, herbaceous flowering plant which originates from Southeast Asia and now extensively cultivated in most tropical and subtropical countries including African countries like Burkina Faso, Cameroon, Ghana, Madagascar, Nigeria, Senegal, and Tanzania. All parts of the plant, especially the rhizome are used in African traditional medicine for the treatment of various conditions like indigestion, gastric ulcerations, constipation, nausea, vomiting, arthritis, rheumatism, pains, fever, cough and cold, sore throats, lung diseases, cramps, hypertension, infectious diseases, asthma, and diabetes [160, 161].

*Zingiber officinale* possesses high pharmacological activities such as antioxidant, anti-microbial, anti-inflammatory, anti-arthritic, anti-platelet, anti-rhinoviral, cardiovascular protection, glucose lowering, and anti-cancer activities [158, 162, 163].

Several studies on *Zingiber officinale* as a potential inhibitor of infections from coronaviruses have been reported in literature [126, 164, 165]. It was recommended as a component of a formulation for the treatment of SARS-CoV-2 [166, 167].

## Conclusion

**Fig. 1:**
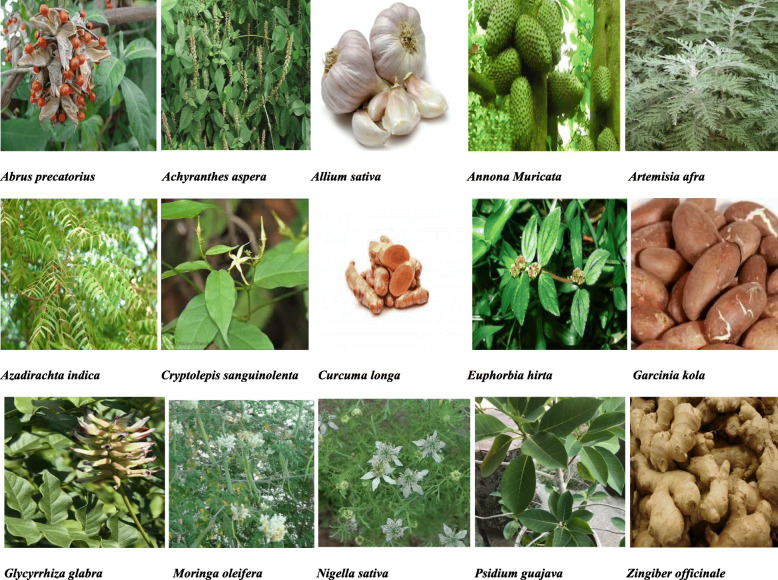
Photos of ethnomedicinal herbs with potential activity for the management of coronavirus disease 2019

Drug discovery from herbs is of great importance which should be exploited for the discovery of drugs for the management of COVID-19. In this review, fifteen (15) ethnomedicinal herbs used in African traditional medicine from different countries in Africa which may be valuable in the prevention, treatment and management of coronavirus disease 2019 were identified (Table 1 and Fig. 1). Due to the complex nature of SARS-CoV-2 and clinical presentation of COVID-19 disease, combining two or more extracts with various pharmacological activity from these herbs in a standard dosage form such as capsule, tablets, syrups, and injections is necessary in the management of the disease. This combination would improve adherence but care must be taken to ensure that all ingredients in the formulation are compatible otherwise it may lead to therapeutic failure or toxicity.

**Table 1:** Summary of the identified ethnomedicinal herbs

S/N	Herb	Constituent	Pharmacological activity	Reference
1.	*Abrus precatorius* Linn.	Abrine, Abrectorin, Abrusoside A, Abraline	Anti-convulsant, anti-asthmatic**,** anti-inflammatory, anti-viral	[15, 19–22]
2.	*Achyranthes aspera* Linn.	Ecdysterone, Eugenol, Betaine, Triacontanol	Anti-inflammatory, antioxidant	[29, 33, 34]
3.	*Allium sativum* Linn.	Ajoene, Allicin, Diallyl disulfide, Vinyldithiins	Anti-inflammatory, antioxidant, anti-viral, anti-cancer	[39, 40, 42, 43]
4.	*Annona muricata* Linn.	Annocatalin, Annomuricin A, Annocatacin, Muricatocin	Anti-inflammatory, anti-microbial, anti-viral	[51, 52, 54]
5.	*Artemisia afra* Jacq.	Acacetin, Scopoletin, Yomogiartemin	Anti-asthmatic, anti-malarial	[58–60]
6.	*Azadirachta indica* A. Juss.	Azadirachtin, Nimbin, Nimbolide, Nimbidin	Anti-malarial, anti-bacterial, anti-fungal, anti-viral	[68, 71–73]
7.	*Cryptolepis sanguinolenta* Lindl.	Cryptolepicarboline, Cryptolepine, Cryptospirolepine	Anti-inflammatory, anti-cancer anti-viral, anti-hyperglycemic	[81, 84, 85]
8.	*Curcuma longa* Linn.	Curcumin, Quercetin, Curcuminoids	Anti-inflammatory, antioxidant, anti-diabetic, anti-coagulant, anti-microbial, anti-viral	[89, 90, 92, 93]
9.	*Euphorbia hirta* Linn.	Rutin, Euphorbin E, Kaempferol, Afzelin	Anti-malarial, anthelmintic, anti-asthmatic, anti-spasmodic, anti-bacterial, anti-fungal, anti-viral	[99–101]
10.	*Garcinia kola* Heckel	Kolanone, Kolaflavanone, Garcinoic acid, Kolaviron	Anti-oxidant, anti-asthma, anti-arthritis, anti-hypertensive, anti-microbial, anti-viral	[105, 106, 109]
11.	G*lycyrrhiza glabra* Linn.	Glycyrrhizin, Licoriphenone, Glycyrrhizic acid	Anti-inflammatory, antioxidant, anti-allergic, anti-cancer, anti-microbial, anti-viral	[114, 116, 117]
12.	*Moringa oleifera* Lam.	Apigenin, Luteolin, Phytosterols, Quercetin	Anti-inflammatory, antioxidant, anti-allergic, anti-microbial, anti-cancer, anti-ulcer, anti-viral	[124–126, 129, 130]
13.	*Nigella sativa* Linn.	Thymoquinone, Cymene, Carvacrol, Thymohydroquinone	Anti-inflammatory, antioxidant, anti-cancer, analgesic, anti-viral anti-microbial, anti-parasitic	[137, 139, 140]
14.	*Psidium guajava* Linn.	Ascorbic acid, Caryophyllene, Gallic acid	Anti-inflammatory, antioxidant, anti-microbial, anti-cancer, immune-system stimulatory	[147, 151]
15.	*Zingiber officinale* Roscoe	Zingiberene, Zingerone, Gingerol, Gingerdiol	Anti-inflammatory, antioxidant, anti-microbial, anti-rhinoviral, anti-cancer	[158, 159, 162, 163]

In conclusion, this review will serve as a source of information for future research in the selection of herbs which could be used in the management of COVID-19. However, experimental analyses and clinical studies would be required for validation.

## Data Availability

Data sharing is not applicable to this article as no datasets were generated or analyzed during the current study.
